# Emerging research themes in ferroptosis research for non-small cell lung cancer: a bibliometric and visualized analysis

**DOI:** 10.3389/fimmu.2025.1563108

**Published:** 2025-05-16

**Authors:** Wenbo Zhang, Jianwei Gu, Yong Chen, Guolu Jiang, Diego Gonzalez-Rivas, Minjie Ma, Chang Chen

**Affiliations:** ^1^ School of Traditional Chinese and Western Medicine, Gansu University of Chinese Medicine, Lanzhou, China; ^2^ Department of Pulmonary and Critical Care Medicine, Affiliated Hospital of North Sichuan Medical College, Nanchong, China; ^3^ Department of Thoracic Surgery, The First Hospital of Lanzhou University, Lanzhou, China; ^4^ Department of Thoracic Surgery, Shanghai Pulmonary Hospital, Shanghai, China; ^5^ Department of Thoracic Surgery and Minimally Invasive Thoracic Surgery Unit (UCTMI), Coruña University Hospital, A Coruña, Spain; ^6^ Shanghai Engineering Research Center of Lung Transplantation, Shanghai, China

**Keywords:** ferroptosis, non-small cell lung cancer, bibliometrics, immunotherapy, biomarkers, drug resistance, nanomedicine

## Abstract

**Background:**

Ferroptosis, an iron-dependent form of regulated cell death, has garnered significant attention as a potential therapeutic target in oncology due to its unique mechanism involving lipid peroxidation and reactive oxygen species accumulation. In non-small cell lung cancer (NSCLC), ferroptosis offers promising strategies to overcome drug resistance and enhance the efficacy of existing therapies. While the literature on ferroptosis in NSCLC has expanded rapidly over the past decade, a comprehensive understanding of its research trends, global collaboration patterns, and emerging hotspots remains lacking.

**Objective:**

This study employs bibliometric and visualized analysis to systematically evaluate global research trends, influential contributors, and thematic evolution in ferroptosis research for NSCLC. The findings aim to guide future investigations and promote interdisciplinary collaborations.

**Methods:**

Data were extracted from the Web of Science Core Collection on December 24, 2024. Bibliometric tools including VOSviewer, CiteSpace, and GraphPad Prism were used to analyze publication trends, citation patterns, collaborative networks, and research hotspots. Key indicators such as publication output, geographic contributions, institutional performance, and keyword co-occurrence were visualized to elucidate the field’s development.

**Results:**

A total of 964 publications from 52 countries and regions were analyzed, with China and the United States emerging as the most influential contributors. Chinese institutions such as Fudan University and Central South University led in publication output, while US-based authors had the highest citation impact. Research hotspots included ferroptosis mechanisms, biomarkers, oxidative stress, immunotherapy, and drug resistance. Keyword and citation analyses reveal an increasing emphasis on integrating ferroptosis inducers with immune checkpoint inhibitors and leveraging nanomedicine for targeted therapy.

**Conclusion:**

This bibliometric analysis highlights the rapid expansion of ferroptosis research in NSCLC, revealing key contributors, global trends, and emerging areas of focus. The integration of ferroptosis with immunotherapy and precision medicine holds immense promise for advancing NSCLC treatment. Future research should prioritize international collaboration, explore resistance mechanisms, and harness advanced technologies such as nanomedicine and artificial intelligence to maximize therapeutic potential.

## 1 Introduction

Ferroptosis, a recently identified form of regulated cell death characterized by iron-dependent lipid peroxidation, has emerged as a promising area of investigation in oncology (1). Unlike apoptosis, necrosis, and autophagy, ferroptosis is distinguished by its reliance on intracellular iron and the accumulation of reactive oxygen species (ROS) derived from lipid peroxides, making it a unique mechanism of cell death (2, 3). In cancer research, ferroptosis has garnered increasing attention due to its potential to overcome drug resistance and enhance the efficacy of existing treatments (4, 5). Lung cancer, a leading cause of cancer-related mortality worldwide, comprises two major types: small cell lung cancer (SCLC) and non-small cell lung cancer (NSCLC), with the latter accounting for approximately 85% of cases (6, 7). Despite advancements in targeted therapies and immunotherapies, NSCLC remains a clinical challenge, with high rates of recurrence and metastasis contributing to poor prognoses in many patients (8, 9).

Recent studies have highlighted the role of ferroptosis in regulating lung cancer progression through mechanisms involving oxidative stress, iron metabolism, and interactions within the tumor microenvironment (10). Notably, ferroptosis intersects with cancer immunology by triggering immunogenic cell death, which activates antitumor immunity, and by altering the tumor microenvironment to enhance immunotherapy efficacy. By inducing ferroptosis, researchers aim to develop novel therapeutic strategies that target resistant tumor cells, enhance the cytotoxic effects of chemotherapy and radiotherapy, and modulate immune responses (10, 11). The combination of ferroptosis inducers with immune checkpoint inhibitors has shown promising preclinical results in NSCLC models, indicating that ferroptosis could enhance NSCLC immunotherapy strategies (12, 13). However, the interplay between ferroptosis and cancer biology remains complex, with several unresolved questions regarding its regulatory pathways, tumor-specific effects, and potential side effects in clinical applications.

Bibliometric analysis provides a systematic approach to evaluating the progress and dynamics of research fields by analyzing publication data, including authorship, collaboration networks, citation patterns, and keyword clusters (14). As a quantitative and visual tool, bibliometric analysis allows researchers to identify influential studies, emerging hotspots, and global trends, offering valuable insights for guiding future research. While the literature on ferroptosis in lung cancer has expanded rapidly over the past decade, a comprehensive bibliometric review of this field has yet to be conducted.

This study aims to address this gap by systematically analyzing publications related to ferroptosis in NSCLC. By examining trends in publication output, geographic distribution, institutional collaborations, and research hotspots, we seek to provide a comprehensive overview of the field’s development. By providing a detailed overview of the current state of ferroptosis research in lung cancer, this study seeks to inform interdisciplinary collaborations, foster innovation, and contribute to the translation of ferroptosis-based therapies into clinical practice.

## 2 Materials and methods

### 2.1 Data acquisition

**Figure 1:**
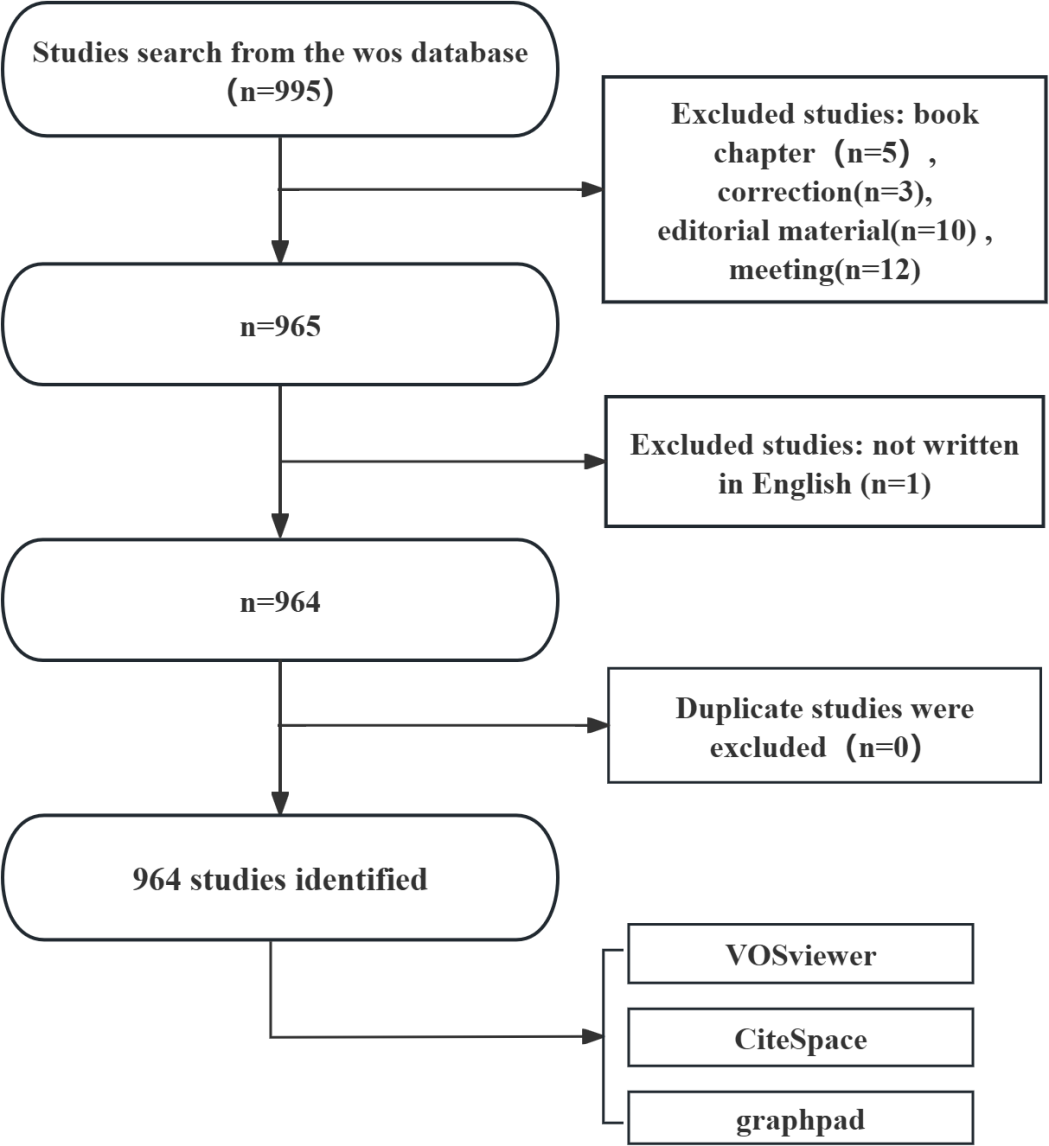
Flowchart of literature search.

Web of Science Core Collection (WoSCC), as a high-quality digital literature resource database encompassing various fields, has been widely accepted by researchers and is considered the optimal choice for conducting bibliometric analysis (14, 15). WoSCC was selected as the primary data source due to its extensive coverage of high-impact, peer-reviewed journals, which ensures the inclusion of rigorously vetted research in ferroptosis and NSCLC. This focus on high-quality publications aligns with the study’s goal of providing a reliable and comprehensive analysis of the field. On December 24, 2024, we conducted a search for all articles related to ferroptosis in lung cancer within WoSCC, using the following search query: (((((((((((TS=("Carcinoma, Non-Small-Cell Lung")) OR TS=("Carcinoma, Non Small Cell Lung")) OR TS=("Carcinomas, Non-Small-Cell Lung")) OR TS=("Lung Carcinoma, Non-Small-Cell")) OR TS=("Lung Carcinomas, Non-Small-Cell")) OR TS=("Non-Small-Cell Lung Carcinomas")) OR TS=("Carcinoma, Non-Small Cell Lung")) OR TS=("Non-Small Cell Lung Cancer")) OR TS=("Non-Small-Cell Lung Carcinoma")) OR TS=("Non Small Cell Lung Carcinoma")) OR TS=("Nonsmall Cell Lung Cancer")) OR TS=("Non-Small Cell Lung Carcinoma")) OR TS=(NSCLC) AND TS=(Ferroptosis). The literature selection for this study was based on the following inclusion criteria: (1) full-text publications related to ferroptosis in lung cancer; (2) articles and review manuscripts written in English. The exclusion criteria were as follows: (1) subjects unrelated to ferroptosis in lung cancer; (2) articles in the form of conference abstracts, news, brief reports, etc. The pure text version of the selected papers was exported. The research flow chart is shown in **Figure 1**.

### 2.2 Data analysis and visualization

This study utilized several software tools for data analysis and visualization, including GraphPad Prism (version 8.0.2), CiteSpace (6.2.4R 64-bit Advanced Edition), and VOSviewer (version 1.6.18), to comprehensively analyze the annual publication volume, national publication trends, and various proportions, as well as to generate scientific knowledge maps. GraphPad Prism v8.0.2 was used to calculate and display the annual publication volume, publication trends, and research proportions across different regions/countries (16). CiteSpace, developed by Professor Chaomei Chen, is a literature analysis software mainly used for visualizing and constructing co-citation networks (17). By creating an experimental framework, CiteSpace aids researchers in identifying emerging concepts within a specific field and assessing existing research techniques. Additionally, CiteSpace is capable of revealing frontier issues and development trends in research, as well as forecasting future academic progress. VOSviewer, a free Java-based software developed by Waltman et al., is designed for analyzing large-scale bibliometric data and visualizing the results (18). The software offers several visualization modes, including network visualization, overlay visualization, and density visualization, which assist users in effectively identifying collaboration patterns, co-occurring terms, and research hotspots in the literature. In VOSviewer, the minimum threshold for keyword co-occurrence was set to 8, with the default 'association strength' algorithm applied, the layout configured to its default values, a cluster resolution of 1, and the 'merge small clusters' option selected. In CiteSpace, the analysis spanned 2015 to 2024, with a time slice of 1 year per segment, using the default term source and analyzing one item at a time; the selection criteria utilized the g-index with a k-value of 25, while other parameters remained at their default settings. For clustering, an 'all in one' approach was adopted using the 'cluster' option, with layout and style optimized for clarity, and clustering was based on 'Keywords' as labels, employing the log-likelihood ratio (LLR) method. For citation burst analysis, the γ value was set to 0.1, the minimum duration to 1 year, with all other settings kept default, and the top 50 burst keywords were selected for further examination. These tools provided multidimensional support for data analysis and visualization in this study, helping to identify and assess the patterns, trends, and collaborations within the literature, as well as revealing the key issues and future directions of research in the field. This study followed the guidelines for reporting bibliometric reviews of biomedical literature (BIBLIO) (19).

## 3 Results

### 3.1 Global trend in publication outputs and citations

**Figure 2:**
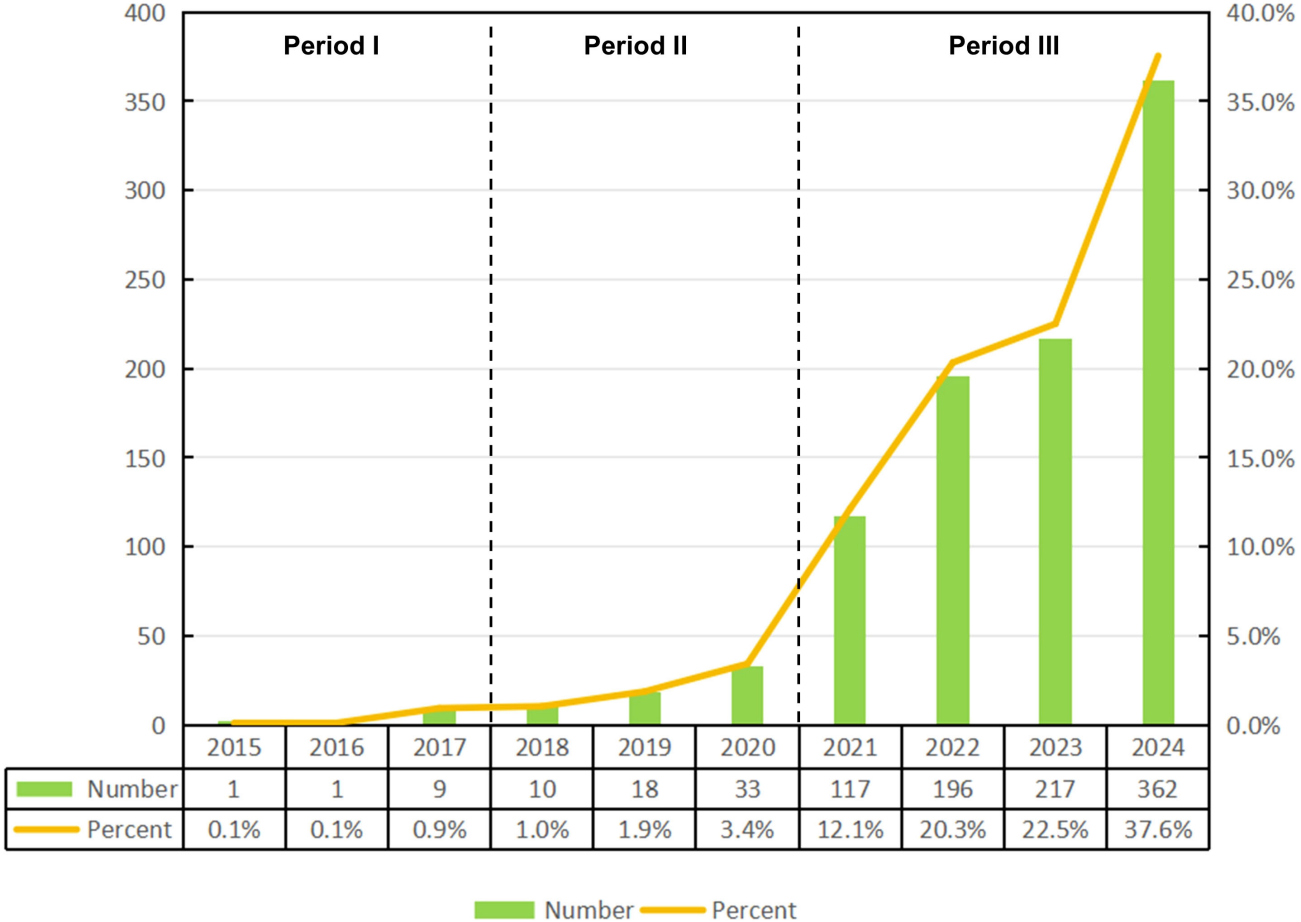
Published trend chart concerning ferroptosis research for non-small cell lung cancer.

The analysis revealed that, as of December 24, 2024, a total of 964 publications related to ferroptosis in lung cancer were indexed in the WoSCC database, comprising 757 original articles and 207 reviews. These studies were contributed by researchers from 52 countries and regions, 1,157 institutions, and 5,198 authors. As shown in **Figure 2**, the annual publication output and citation frequency of related studies from 2015 to 2024 exhibited a clear growth trend, which can be divided into three phases: before 2017, the annual output remained below 10 publications; since 2018, the annual output exceeded 10 publications, marking a steady increase; and after 2021, the annual output surpassed 100 publications for the first time, reaching 117, and continued to grow significantly, peaking at 362 publications in 2024. This rapid growth in publication volume indicates that ferroptosis in lung cancer is likely to remain a prominent and active area of research in the foreseeable future.

### 3.2 Distribution of countries/regions

**Figure 3:**
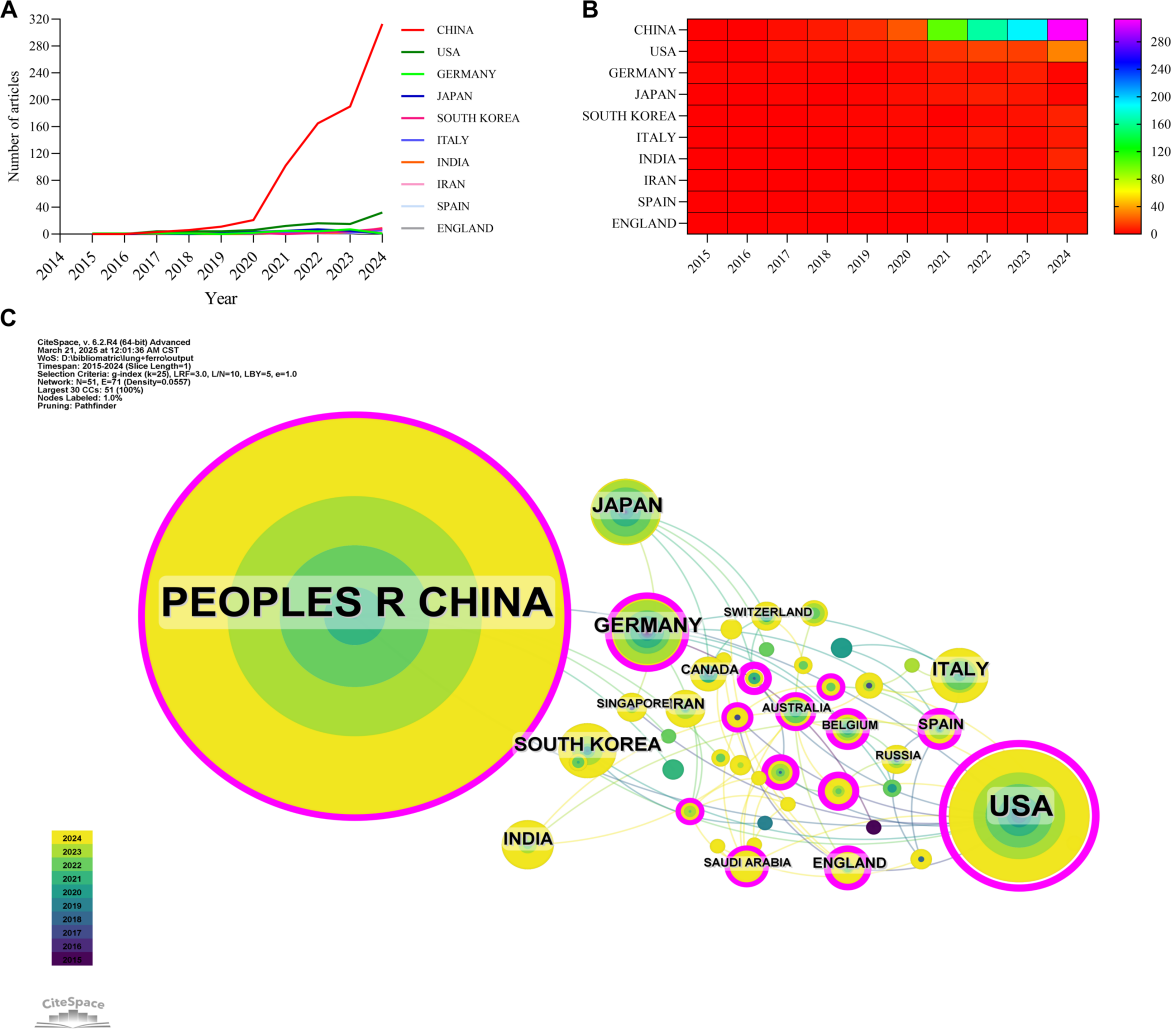
Country/region collaboration network of research on ferroptosis for non-small cell lung cancer. **(A)** Line graph of national publications. **(B)** Heat map of national publications. **(C)** Networks of country cooperation, node size represents the volume of publications and the line color represents the year of collaboration.

A total of 52 countries and regions have conducted research on the application of ferroptosis in lung cancer. **Figures 3A**, **B** display the annual publication output of the top 10 countries over the past decade. The top five countries in this field are China, the United States, Germany, Japan, and South Korea. China accounts for 84.13% of the total publications, significantly outpacing all other countries. Among the top 10 countries/regions in terms of publication output, China’s papers have been cited 20,915 times (**Table 1**), far exceeding all other nations. Its citation/publication ratio (25.79) ranks fourth globally, indicating that the quality of its publications is generally high. The United States ranks second in publication output (93 papers) and second in citations (7,721), with the highest citation/publication ratio (83.02), indicating a strong citation impact. The collaboration network, shown in **Figure 3C**, illustrates close cooperation between China and the United States, the two countries with the highest publication output. The United States has strong collaborations with the United Kingdom, Italy, and Spain, while China collaborates more closely with Germany, India, and South Korea. China not only leads in publication volume and citation frequency but also demonstrates exceptional centrality (0.53) in the global scientific collaboration network, indicating its dominant position in this field.

**Table 1:** Table of country published literature.

Rank	Country/region	Article counts	Centrality	Percentage (%)	Citation	Citation per publication
1	CHINA	811	0.53	84.13%	20915	25.79
2	USA	93	0.27	9.65%	7721	83.02
3	GERMANY	23	0.17	2.39%	1349	58.65
4	JAPAN	23	0.09	2.39%	685	29.78
5	SOUTH KOREA	18	0.09	1.87%	351	19.50
6	ITALY	17	0.1	1.76%	383	22.53
7	INDIA	15	0.11	1.56%	105	7.00
8	IRAN	9	0.12	0.93%	88	9.78
9	SPAIN	8	0.07	0.83%	306	38.25
10	ENGLAND	8	0.1	0.83%	148	18.50

### 3.3 Institutions

**Figure 4:**
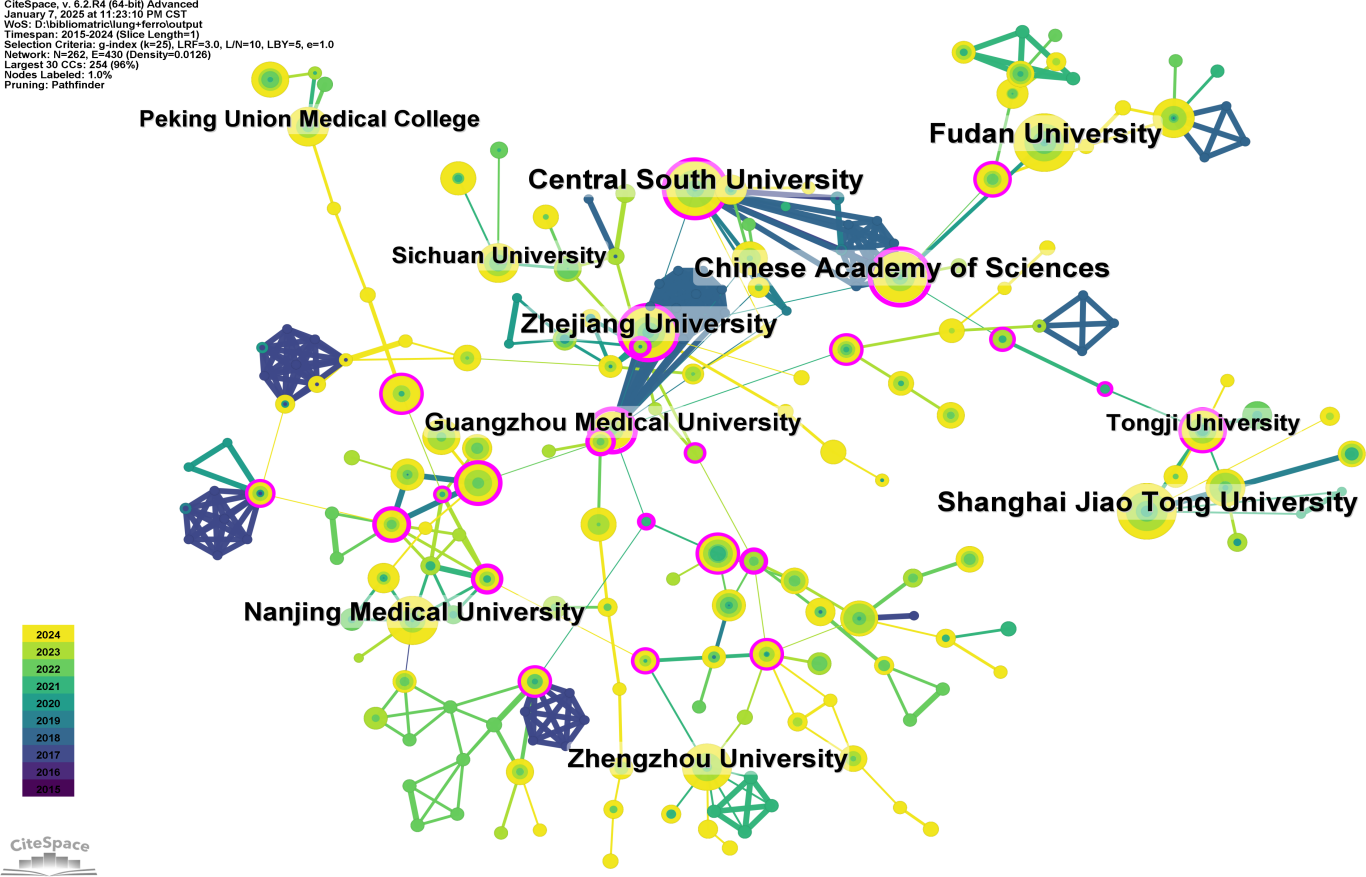
Networks of institutional co-operation.

A total of 1,157 institutions have systematically published research related to ferroptosis in lung cancer. The top ten institutions in terms of publication output are all from China (**Table 2**, **Figure 4**). Fudan University leads with the highest number of publications (44 papers, 2,040 citations, an average of 46.36 citations per paper). Central South University ranks second (42 papers, 2,067 citations, an average of 49.21 citations per paper), followed by Shanghai Jiao Tong University (41 papers, 922 citations, an average of 22.49 citations per paper) in third place, and the Chinese Academy of Sciences (39 papers, 1,922 citations, an average of 49.28 citations per paper) in fourth place. Further analysis revealed that both domestic and international institutions tend to collaborate more frequently with institutions within their own countries. This underscores the need to strengthen international collaborations and break down academic barriers.

**Table 2:** Table of Institutional Published Literature.

Rank	Institution	Country	Number of studies	Total citations	Average citation
1	Fudan University	China	44	2040	46.36
2	Central South University	China	42	2067	49.21
3	Shanghai Jiao Tong University	China	41	922	22.49
4	Chinese Academy of Sciences	China	39	1922	49.28
5	Zhejiang University	China	35	1832	52.34
6	Nanjing Medical University	China	32	1261	39.41
7	Zhengzhou University	China	30	1559	51.97
8	Guangzhou Medical University	China	26	1266	48.69
9	Tongji University	China	21	1022	48.67
10	Peking Union Medical College	China	21	1073	51.10

### 3.4 Journals

**Figure 5:**
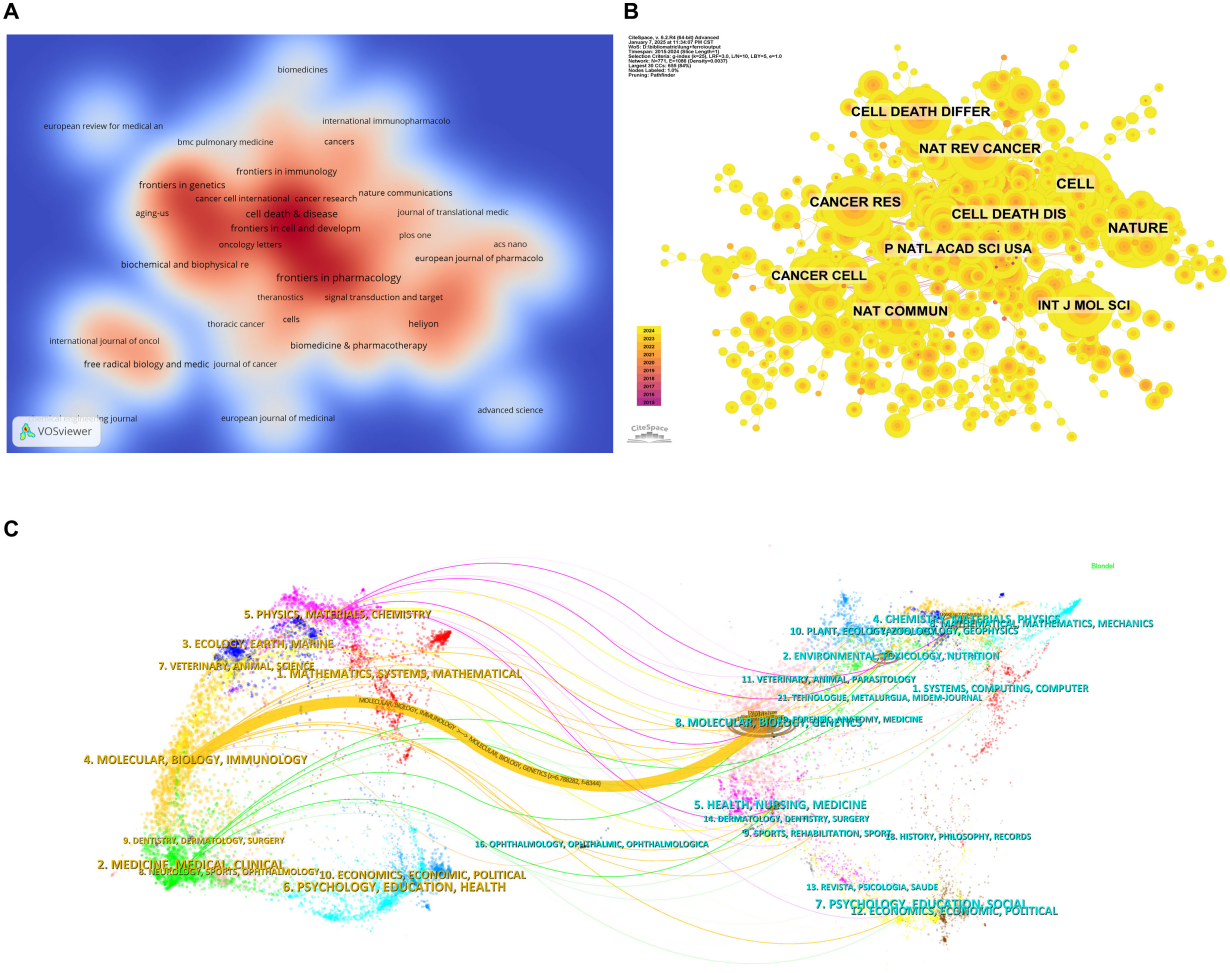
Analysis of journal sources. **(A)** Density web map of journal publications. **(B)** Co-citation network map of journals. **(C)** Dual map of journals.

**Tables 3** and **4** list the top 10 journals in terms of both publication output and citation frequency. Frontiers in Pharmacology (28 papers, 2.90%) is the most prolific journal in this field, followed by Frontiers in Oncology (26 papers, 2.70%), Cell Death & Disease (22 papers, 2.28%), and Frontiers in Cell and Developmental Biology (18 papers, 1.87%). Among the top ten most prolific journals, Cell Death & Disease has the highest impact factor (IF) of 8.1. All of the journals listed fall within the Q1 or Q2 quartiles. The elevated proportion of Q1 journals could be attributed to the field's inclination toward high-impact, stringently peer-reviewed publications, which correspond closely with the research topic's significance and interdisciplinary character. The journal impact is determined by its citation frequency, indicating the significant influence the journal has on the scientific community. According to the density map of the journals, publications in this field primarily fall into four categories: oncology, immunology, pharmacology, and comprehensive (**Figure 5A**).

**Table 3:** Table of Journal Publications.

Rank	Journal	Article counts	Percentage (964)	IF	Quartile in category
1	frontiers in pharmacology	28	2.90%	4.4	Q1
2	frontiers in oncology	26	2.70%	3.5	Q2
3	cell death & disease	22	2.28%	8.1	Q1
4	frontiers in cell and developmental biology	18	1.87%	4.6	Q1
5	cell death discovery	17	1.76%	6.1	Q1
6	frontiers in genetics	16	1.66%	2.8	Q2
7	biomedicine & pharmacotherapy	13	1.35%	6.9	Q1
8	frontiers in molecular biosciences	13	1.35%	3.9	Q2
9	international journal of molecular sciences	13	1.35%	4.9	Q1
10	frontiers in immunology	12	1.24%	5.7	Q1

**Table 4:** Co-citation table of journals.

Rank	Cited Journal	Co-Citation	IF(2023)	Quartile in category
1	CELL	728	45.6	Q1
2	NATURE	666	50.5	Q1
3	CELL DEATH DIS	562	8.1	Q1
4	CANCER RES	523	12.5	Q1
5	NAT COMMUN	494	14.7	Q1
6	CELL DEATH DIFFER	476	13.7	Q1
7	NAT REV CANCER	434	72.5	Q1
8	P NATL ACAD SCI USA	430	9.4	Q1
9	CANCER CELL	422	48.8	Q1
10	INT J MOL SCI	420	4.9	Q1

Based on **Figure 5B** and **Table 4**, the most frequently co-cited journal is CELL (728 citations), followed by NATURE (666 citations) and Cell Death & Disease (562 citations). Among the top ten most co-cited journals, Nature Reviews Cancer (NAT REV CANCER) is the most highly cited (434 citations), with the highest IF (72.5) among the top ten. All co-cited journals fall within the Q1 quartile. The thematic distribution of academic publications is illustrated through the dual-map overlay (**Figure 5C**), where the colored trajectories represent citation links. The citing journals are on the left, and the cited journals are on the right. From the results, we identified a major citation pathway: research published in journals within the Molecular/Biology/Genetics domain is predominantly cited by research published in journals within the Molecular/Biology/Immunology domain.

### 3.5 Authors

**Figure 6:**
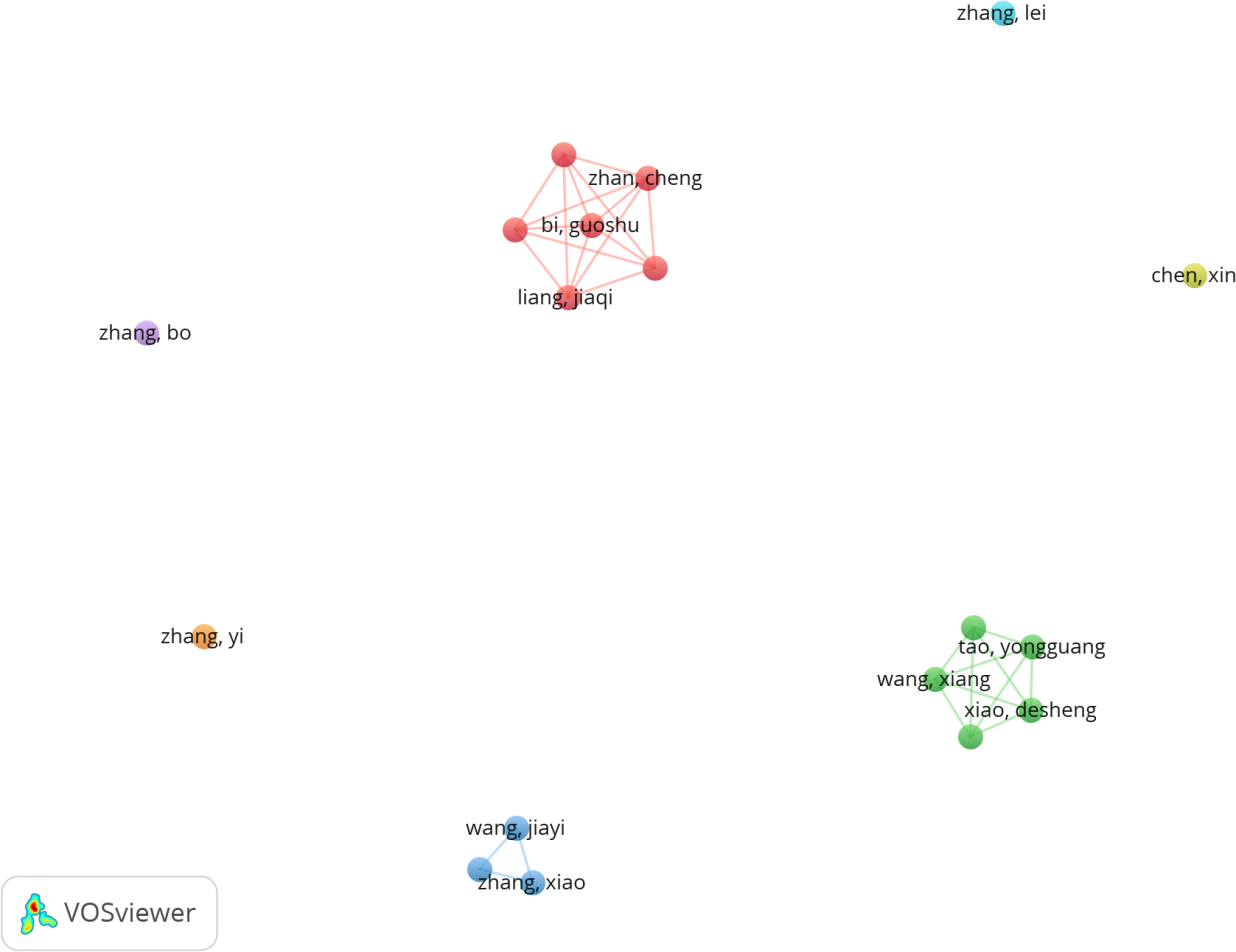
Cooperation network of authors.

**Figure 7:**
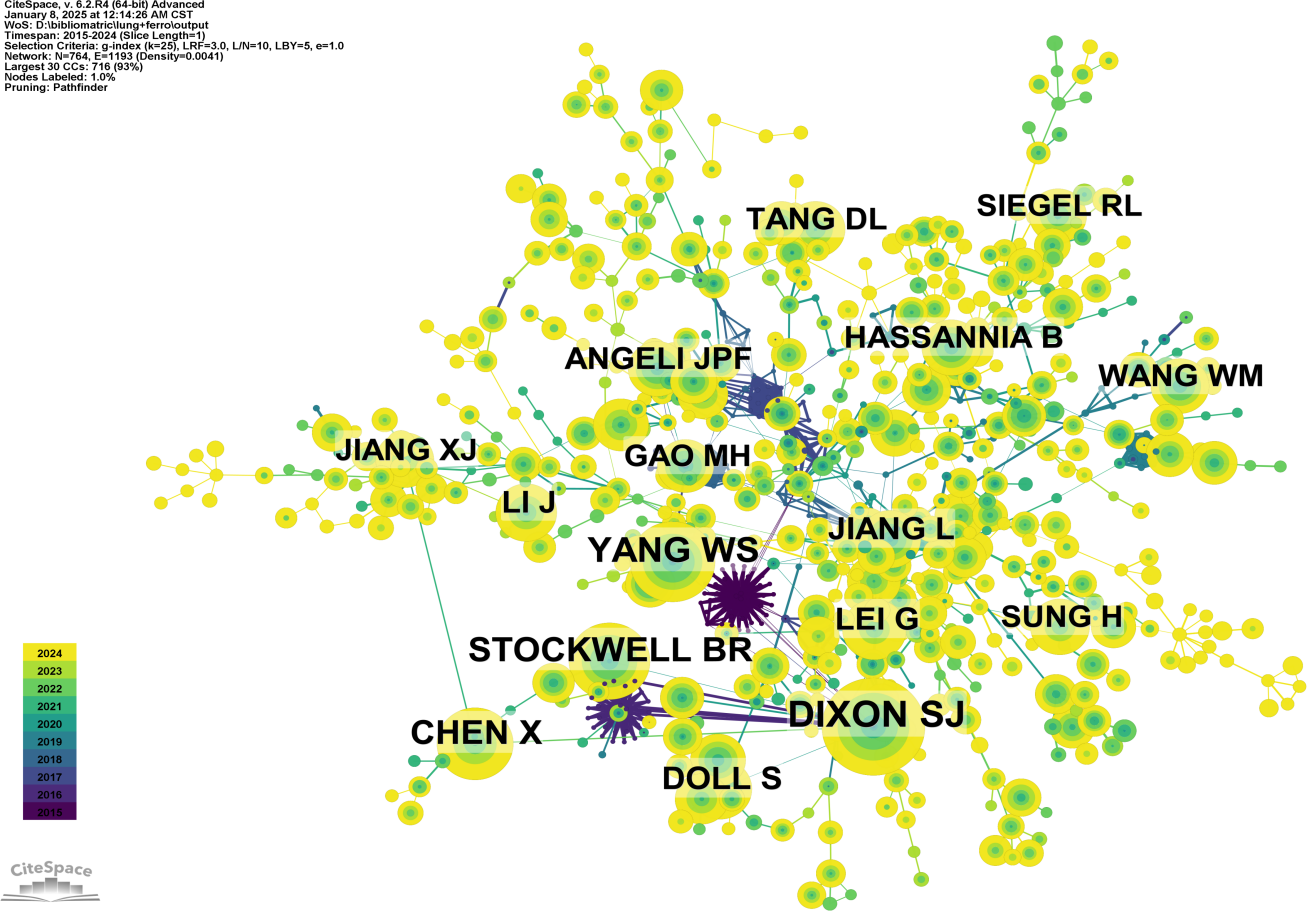
Co-citation network of authors.

Among all authors who have published research on ferroptosis in lung cancer, **Table 5** lists the top 10 most prolific authors. These authors collectively contributed 87 papers, accounting for 7.04% of all publications in this field. Tao Yongguang authored the most papers (10), followed by Zhan Cheng (10), Liu Shuang (9), Wang Xiang (9), and Zhang Xiao (9). The collaborative network among authors was visualized using CiteSpace (**Figure 6**). **Figure 7** and **Table 5** present the top 10 most frequently co-cited and cited authors, respectively. A total of 86 authors were cited more than 50 times, highlighting their significant reputation and influence in the field. The largest nodes in the co-citation network correspond to the most frequently co-cited authors, including Dixon SJ (504 citations), Yang WS (343 citations), and Stockwell BR (310 citations). Tao Yongguang and Dixon SJ are pioneers in applied research and basic research, respectively. Tao's series of work focused on the therapeutic potential of ferroptosis in NSCLC, while Dixon's pioneering research first defined ferroptosis and laid the theoretical foundation for this field.

**Table 5:** Author's publications and co-citation table.

Rank	Author	Count	Rank	Co-cited author	Citation
1	Tao, yongguang	10	1	Dixon sj	504
2	Zhan, cheng	10	2	Yang ws	343
3	Liu, shuang	9	3	Stockwell br	310
4	Wang, xiang	9	4	Chen x	272
5	Zhang, xiao	9	5	Doll s	198
6	Bi, guoshu	8	6	Lei g	187
7	Liang, jiaqi	8	7	Jiang l	184
8	Ma, lifang	8	8	Li j	174
9	Xiao, desheng	8	9	Hassannia b	171
10	Zhang, bo	8	10	Angeli jpf	164

### 3.6 Citation and co-citation analysis

Citation analysis is a key method for assessing the relevance of academic papers, as it reflects both the influence of articles within a field and the research hotspots in that domain. **Table 6** lists the top 10 most cited papers. The most cited paper, titled "CD8+ T cells regulate tumour ferroptosis during cancer immunotherapy," has been cited 161 times, elucidating the synergistic interplay between ferroptosis and antitumor immunity. This is followed by ""Ferroptosis: mechanisms, biology and role in disease," with 154 citations, and "Broadening horizons: the role of ferroptosis in cancer" with 152 citations, which collectively provide a systematic summary of the molecular mechanisms of ferroptosis, offering a theoretical framework for identifying ferroptosis-related targets (20–22).

**Table 6:** Co-citation table of literature.

Rank	Title	Journal	Author(s)	Total citations
1	CD8+ T cells regulate tumour ferroptosis during cancer immunotherapy	NATURE	Wang WM	161
2	Ferroptosis: mechanisms, biology and role in disease	NAT REV MOL CELL BIO	Jiang XJ	154
3	Broadening horizons: the role of ferroptosis in cancer	NAT REV CLIN ONCOL	Chen X	152
4	The CoQ oxidoreductase FSP1 acts parallel to GPX4 to inhibit ferroptosis	NATURE	Bersuker K	149
5	Targeting Ferroptosis to Iron Out Cancer	CANCER CELL	Hassannia B	148
6	FSP1 is a glutathione-independent ferroptosis suppressor	NATURE	Doll S	139
7	Ferroptosis: A Regulated Cell Death Nexus Linking Metabolism, Redox Biology, and Disease	CELL	Stockwell BR	133
8	Ferroptosis: past, present and future	CELL DEATH DIS	Li J	107
9	Targeting ferroptosis as a vulnerability in cancer	NAT REV CANCER	Lei G	105
10	Ferroptosis: molecular mechanisms and health implications	CELL RES	Tang DL	104

**Figure 8:**
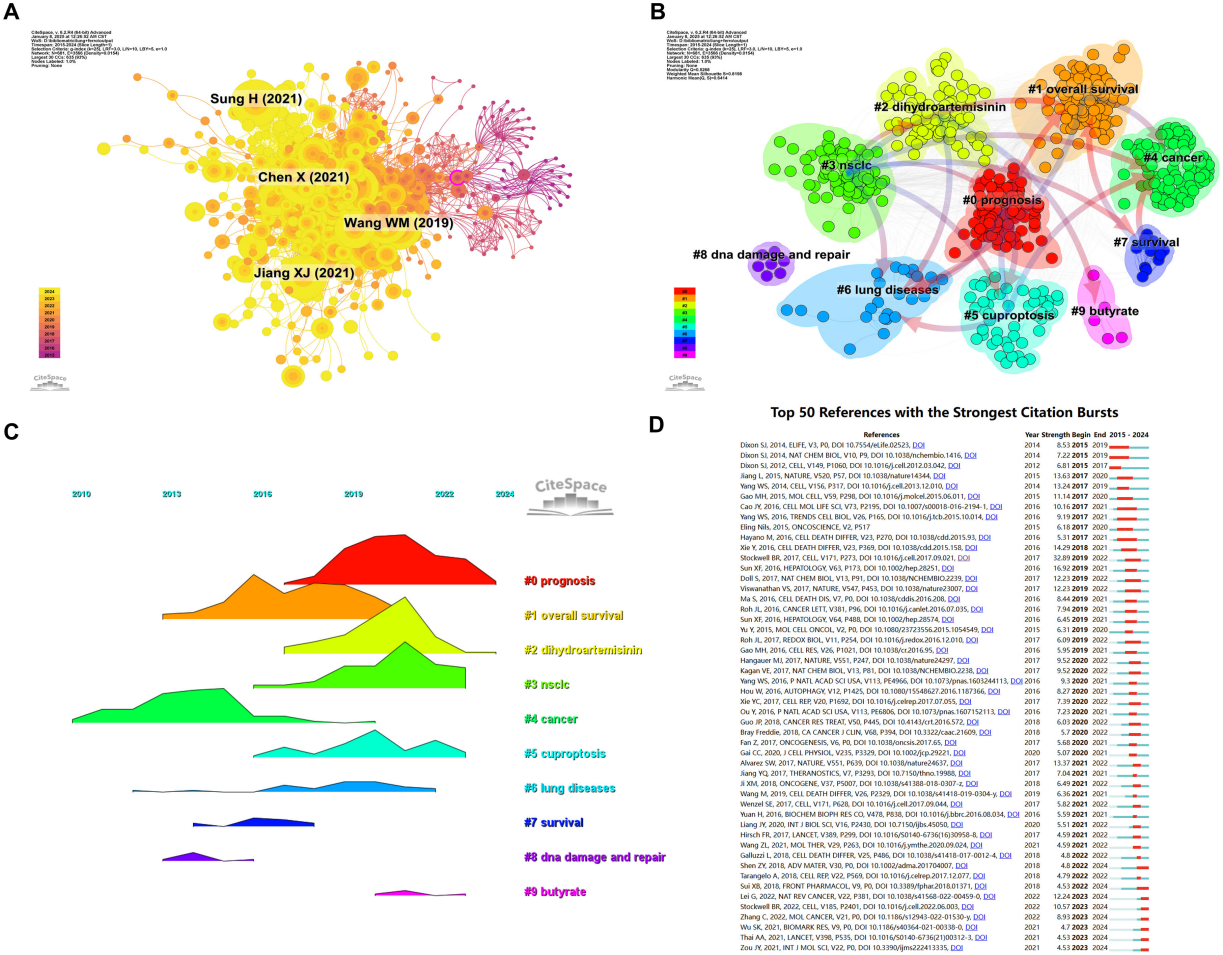
Analysis cited references and co-cited references. **(A)** Co-cited network of literature. **(B)** Clustering of co-cited literature. **(C)** Peak map of co-cited literature. **(D)** Bursting map of cited literature.

To further explore the relationships among these citations, a co-citation network was constructed, using a one-year time slice for the period from 2015 to 2024. The co-citation network contains 681 nodes and 3,586 links (**Figure 8A**). Cluster and temporal analyses of the co-cited references revealed nine distinct clusters (**Figures 8B,C**). Early research hotspots were identified in the cancer cluster (Cluster 4). Mid-stage hotspots include overall survival (Cluster 1), survival (Cluster 7), and DNA damage and repair. Emerging hotspots and trends in this field are represented by clusters such as prognosis (Cluster 0), dihydroartemisinin (Cluster 2), NSCLC (Cluster 3), cuproptosis (Cluster 5), lung disease (Cluster 6), and butyrate (Cluster 9).

**Figure 8D** illustrates the top 50 references with the strongest citation bursts. The most prominent citation burst is associated with the 2014 paper titled "Pharmacological inhibition of cystine-glutamate exchange induces endoplasmic reticulum stress and ferroptosis", which exhibited the strongest burst strength of 8.53, reflecting its lasting influence on the study of ferroptosis mechanisms (23). While recent burst literature (such as Lei G, 2022, Nat Rev Cancer) points to the latest progress in ferroptosis as a vulnerability in cancer treatment (24). Among the 50 references with the strongest citation bursts, 46 were published between 2015 and 2024, indicating that these papers have been frequently cited over the past decade. Notably, 8 of these references are currently in their peak citation period, suggesting that ferroptosis-related research in lung cancer will continue to attract significant attention in the future.

### 3.7 Keywords and hotspots

**Figure 9:**
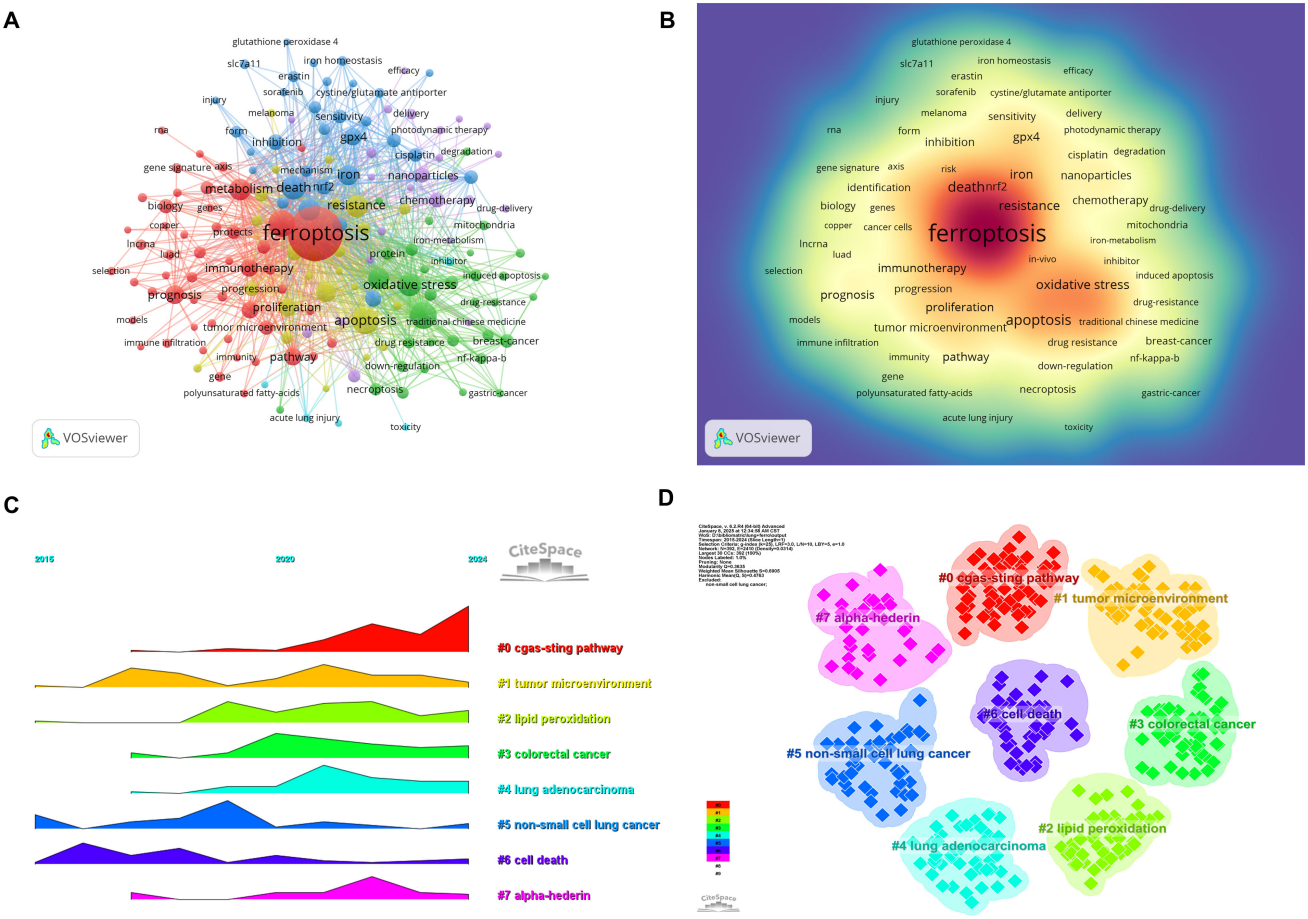
Analysis of keywords associated with ferroptosis in non-small cell lung cancer. **(A)** Network map of high-frequency keywords. **(B)** Density map of keywords. **(C)** Peak map of keyword clustering. **(D)** Clustering map of keywords.

Keyword analysis provides a rapid understanding of the status and developmental trends within a research field. Based on the co-occurrence analysis of keywords in VOSviewer, the most frequently appearing keyword is ferroptosis (681 occurrences), followed by apoptosis (143), cell death (142), lung cancer (140), and death (109) (**Table 7**, **Figures 9A, B**). After removing irrelevant keywords, a network comprising 169 keywords with at least 8 occurrences was constructed, revealing six distinct clusters. Cluster 1 (red) includes 51 keywords, such as ferroptosis, immunotherapy, prognosis, biomarker, and metastasis. Cluster 2 (green) contains 33 keywords, including lung cancer, oxidative stress, autophagy, drug resistance, and tumor suppressor. Cluster 3 (blue) consists of 31 keywords, including iron, GPX4, cisplatin, ROS, and iron metabolism. Cluster 4 (yellow) includes 25 keywords, such as apoptosis, angiogenesis, p53, and invasion. Cluster 5 (purple) comprises 24 keywords, including artemisinin, chemotherapy, drug delivery, and nanomedicine. Finally, Cluster 6 (cyan) contains 5 keywords, including EGFR and toxicity.

**Table 7:** High Frequency Keyword Table.

Rank	Keyword	Counts	Rank	Keyword	Counts
1	ferroptosis	681	11	mechanisms	78
2	apoptosis	143	12	iron	77
3	cell-death	142	13	prognosis	63
4	lung-cancer	140	14	proliferation	60
5	death	109	15	gpx4	55
6	oxidative stress	97	16	immunotherapy	54
7	resistance	92	17	pathway	54
8	metabolism	89	18	metastasis	53
9	activation	84	19	lipid-peroxidation	46
10	autophagy	81	20	inhibition	45

**Figure 10:**
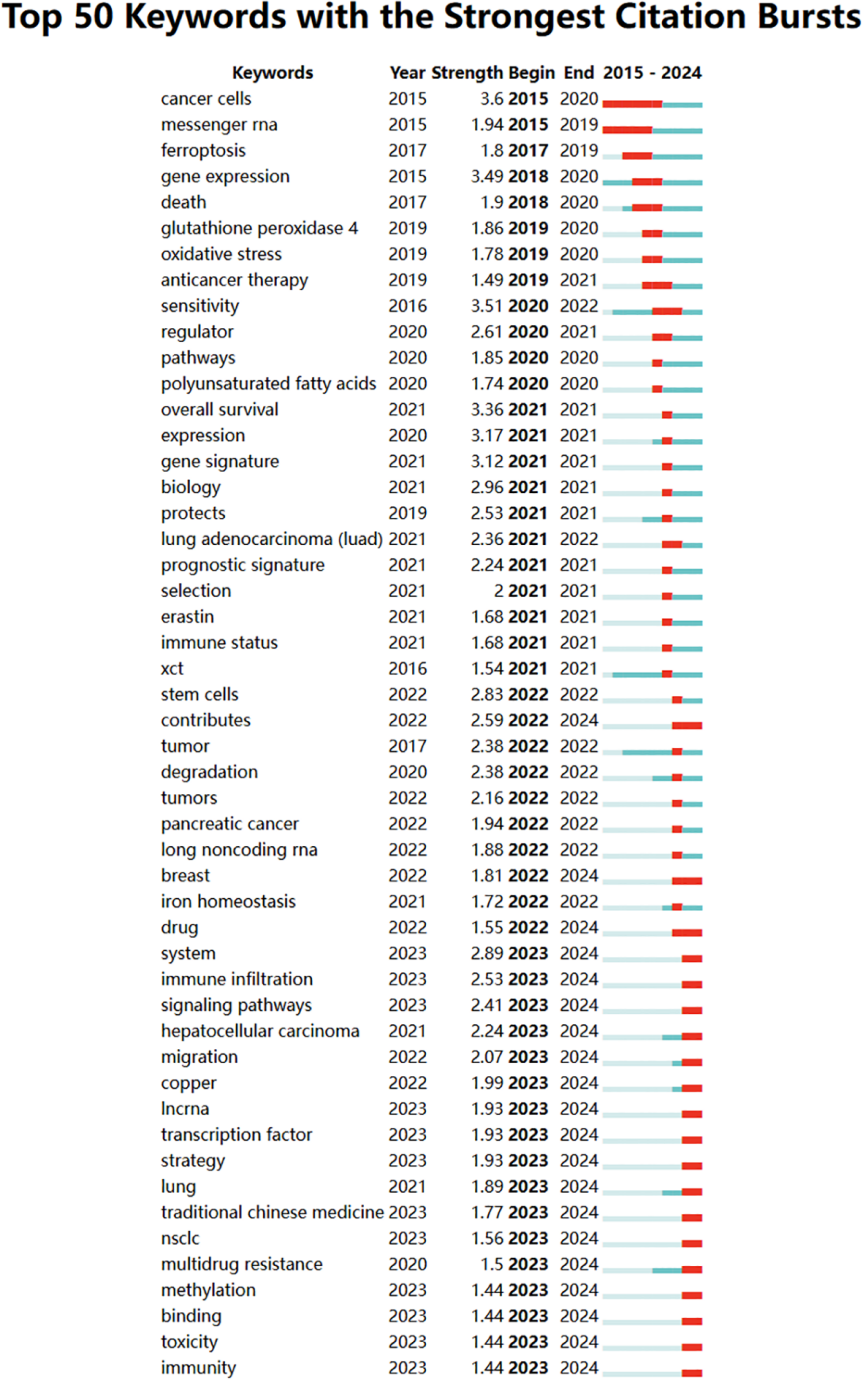
Bursting map of keywords.

A temporal visualization using CiteSpace (**Figures 9C, D**) was generated to illustrate the evolution of research hotspots over time. The analysis revealed that the cGAS-STING pathway, tumor microenvironment, lipid peroxidation, colorectal cancer, lung adenocarcinoma, non-small cell lung cancer, cell death, and alpha hederin are current research hotspots. Among the 434 strongest burst keywords identified in this field, we focused on the top 50 with the strongest citation bursts (**Figure 10**), which represent the current research priorities and potential future directions in this domain.

## 4 Discussion

### 4.1 General information

This bibliometric analysis, diverging from previous pan-cancer bibliometric studies on ferroptosis, centers on NSCLC, offering systematic evaluation of the exponential increase in ferroptosis-related lung cancer research over the past decade, particularly emphasizing the growing integration of ferroptosis with immunotherapy to guide precision medicine advancements. The increasing annual publication output and citation frequency since 2018 reflect a rising global interest in ferroptosis as a promising therapeutic target (25). The growing prominence of ferroptosis aligns with advancements in understanding its unique mechanisms, such as iron-dependent lipid peroxidation, and its potential applications in overcoming therapeutic resistance (21, 26).

The significant contributions of China and the United States to the volume and impact of publications indicate their leadership in ferroptosis research. China, with its extensive research output, has established itself as a dominant force, while the United States leads in citation impact, demonstrating high-quality contributions. This divergence underscores the need for increased international collaborations to enhance both the quantity and quality of research outputs globally (27).

### 4.2 Institutional and author contributions

Institutional analysis reveals that Chinese universities, particularly Fudan University, Central South University, and Shanghai Jiao Tong University, are at the forefront of ferroptosis research, contributing significantly to the field’s publication output. China accounts for 84.13% of total publications, demonstrating its dominance in research volume. However, collaboration networks show a marked preference for domestic partnerships, which may limit the potential for global knowledge sharing. In contrast, the United States, despite a lower publication volume, achieves a significantly higher citation rate (83.02 vs. 25.79), indicating a stronger emphasis on high-quality, impactful research. This disparity may stem from differences in research priorities and funding mechanisms: the United States tends to prioritize original, foundational studies that often lead to paradigm shifts, while China’s rapid publication output may reflect a focus on applied research and translational studies. Additionally, US institutions, such as the University of Texas and Memorial Sloan Kettering Cancer Center, exhibit stronger international partnerships, publishing frequently in high-impact, English-language journals that are widely cited globally, whereas Chinese institutions collaborate more within domestic networks, potentially influenced by language preferences, data sharing policies, and funding constraints (28).

Prominent authors such as Tao Yongguang, Dixon SJ, and Stockwell BR have significantly shaped the field, with research focusing on ferroptosis mechanisms, biomarkers, and therapeutic applications (3, 29). Dixon SJ’s seminal work on the discovery of ferroptosis has catalyzed numerous follow-up studies, positioning him as a key figure in the field, while Stockwell BR’s contributions to understanding ferroptosis regulation have influenced subsequent therapeutic developments (30). The strong influence of these authors underscores the importance of foundational research in driving innovation and collaboration. However, the preference for domestic collaboration in China may be attributed to several factors, including funding mechanisms that incentivize domestic programs, stringent data privacy regulations limiting the exchange of clinical and genomic data critical for ferroptosis research, and challenges related to intellectual property protection. Language barriers and differing research priorities further exacerbate this trend, as US-based research benefits from broader international visibility.

To address these challenges and bridge research gaps, strengthening international collaborations, particularly between leading institutions in China and the United States, is essential. Establishing transnational research alliances could facilitate data sharing, harmonize research protocols, and provide funding for joint projects. Open data platforms compliant with privacy regulations would enable researchers to share datasets securely, while academic exchange program could foster mutual understanding and trust among international teams. Additionally, addressing language barriers through translation services and promoting English as a common scientific language could enhance communication. By promoting these initiatives, the global impact of ferroptosis research could be improved, fostering innovation and accelerating the translation of foundational discoveries into clinical advancements.

### 4.3 Research hotspots and frontiers

Keyword co-occurrence and co-citation analyses reveal distinct clusters representing the major research hotspots in ferroptosis and lung cancer. These include ferroptosis mechanisms, oxidative stress, biomarkers, immunotherapy, and drug resistance. Notably, immunotherapy has emerged as a significant trend, with preclinical studies highlighting the potential of combining ferroptosis inducers with immune checkpoint inhibitors (ICIs) to enhance anti-tumor efficacy (10, 31). Such combinations have shown promise in overcoming immune evasion in NSCLC, underscoring ferroptosis as a complementary strategy in modern oncology immunotherapy (32).

Biomarker identification is another critical research frontier. Markers such as GPX4 and lipid peroxidation pathways are pivotal for predicting ferroptosis susceptibility and tailoring therapeutic interventions (33–35). Advances in nanomedicine, including the development of drug delivery systems targeting tumor-specific ferroptosis pathways, further enhance the precision and efficacy of these treatments (36, 37). Additionally, the cGAS-STING pathway and tumor microenvironment modulation have gained attention for their roles in linking ferroptosis to immune response and inflammation (38).

Temporal analysis of research trends highlights the evolving focus of ferroptosis studies. Early research emphasized fundamental mechanisms and the role of iron metabolism in ferroptosis (39). Mid-stage studies explored therapeutic applications, including chemotherapy and radiotherapy combinations (24, 30). More recently, research has shifted toward integrating precision medicine approaches, such as genomic profiling and personalized therapies, into ferroptosis-based interventions (40–42). This progression reflects the dynamic nature of the field and its responsiveness to technological and clinical advancements.

### 4.4 Ferroptosis mechanisms: an in-depth exploration in lung cancer

Ferroptosis, characterized by iron-dependent lipid peroxidation, is intricately linked to lung cancer progression, particularly in NSCLC. A hallmark of ferroptosis is dysregulated iron metabolism, which contributes to ROS production through the fenton reaction (26). In NSCLC, elevated expression of transferrin receptor 1 (TFR1) and reduced ferroportin levels drive iron accumulation, creating a pro-ferroptotic environment (43). Furthermore, tumor cells often exhibit increased ferritin levels, which confer chemoresistance (44, 45). Strategies targeting ferritinophagy or enhancing iron uptake have shown promise in sensitizing lung cancer cells to ferroptosis. Lipid peroxidation, mediated by enzymes like ACSL4 and lipoxygenases, plays a central role in ferroptosis by oxidizing polyunsaturated fatty acids (PUFAs) in membrane phospholipids (46). The overexpression of ACSL4 in NSCLC enhances ferroptosis susceptibility, while increased mono-unsaturated fatty acid levels confer resistance, highlighting the therapeutic potential of modulating lipid composition in tumors (47).

The glutathione (GSH)-GPX4 axis is another critical regulatory pathway in ferroptosis, with GPX4 reducing lipid hydroperoxides to non-toxic alcohols in a GSH-dependent manner (48). NSCLC cells often rely heavily on this axis to counteract oxidative damage. Inhibitors of GPX4, such as RSL3, and agents targeting cystine uptake, like erastin, disrupt this pathway, leading to ferroptotic cell death (49). The tumor microenvironment (TME) further modulates ferroptosis (50). Hypoxia, commonly observed in lung tumors, inhibits ROS generation, reducing ferroptosis sensitivity. Conversely, reoxygenation or pharmacological normalization of the TME restores ferroptosis susceptibility. The interplay between ferroptosis and immune responses is particularly noteworthy (51). Ferroptotic cancer cells release damage-associated molecular patterns (DAMPs), which activate anti-tumor immunity (52). Combining ferroptosis inducers with immune checkpoint inhibitors has shown synergistic effects in preclinical NSCLC models, highlighting the potential for integrated therapeutic strategies.

Recent advances in genomics and epigenetics provide further insights into ferroptosis regulation in lung cancer. Mutations in key genes, such as KRAS and TP53, alter ferroptosis sensitivity (53). KRAS mutations increase ROS production and ferroptotic vulnerability, while TP53 enhances ferroptosis through the regulation of cystine uptake and glutaminolysis (54). Epigenetic modifications, such as DNA methylation and histone acetylation, also influence ferroptosis-related gene expression (55). Clinically, ferroptosis inducers combined with chemotherapy or immunotherapy offer a promising avenue for overcoming resistance and improving therapeutic efficacy. Nanomedicine, including nanoparticle-based delivery systems, provides a targeted approach to maximize the benefits of ferroptosis inducers while minimizing off-target effects (56).

### 4.5 Clinical translation of ferroptosis mechanisms

The translation of ferroptosis mechanisms into clinical strategies represents a promising frontier in NSCLC treatment. Preclinical studies have demonstrated the efficacy of ferroptosis inducers, such as erastin and RSL3, in sensitizing NSCLC cells to chemotherapy and radiotherapy (57). Notably, the combination of ferroptosis inducers with ICIs has shown synergistic effects in preclinical models, enhancing anti-tumor immunity and overcoming immune evasion. Recent studies have reported that ferroptotic cancer cells release DAMPs, which activate dendritic cells and promote T cell-mediated anti-tumor responses (58).

Despite the promising preclinical results, the clinical translation of ferroptosis research faces several challenges. First, the lack of validated biomarkers for ferroptosis susceptibility poses a significant barrier to patient stratification and treatment optimization. While GPX4 and ACSL4 have emerged as potential biomarkers, their clinical utility remains to be confirmed in large-scale studies. Second, the potential off-target effects of ferroptosis inducers, such as systemic toxicity and damage to normal tissues, raise concerns about their safety in clinical applications. Developing targeted delivery systems, such as nanoparticle-based carriers, could mitigate these risks by selectively delivering ferroptosis inducers to tumor sites. Third, the complex interplay between ferroptosis and the TME complicates its clinical application. For example, hypoxia and nutrient deprivation in the TME can reduce ferroptosis sensitivity, necessitating strategies to modulate the TME for enhanced therapeutic efficacy. Finally, regulatory and logistical challenges, such as the design of robust clinical trials and the establishment of standardized protocols, must be addressed to accelerate the translation of ferroptosis research into clinical practice (59).

### 4.6 Future research trends

The future of ferroptosis research in lung cancer is centered on unraveling its complex molecular mechanisms and integrating this knowledge into precision medicine. While significant strides have been made, further exploration of ferroptosis' interplay with other cell death pathways, such as apoptosis and autophagy, remains essential (60). Investigating the role of ferroptosis within the TME, including its effects on immune cells and stromal interactions, can unveil strategies to modulate the TME for enhanced therapeutic efficacy. Moreover, advances in genomic profiling have identified key biomarkers that influence ferroptosis sensitivity (61). Integrating these biomarkers into clinical workflows will enable the stratification of patients and the personalization of ferroptosis-based therapies. Predictive models that combine genomic, transcriptomic, and metabolic data hold promise for optimizing patient selection and treatment strategies. Nanotechnology-based drug delivery systems, including lipid nanoparticles and polymeric micelles, offer targeted delivery of ferroptosis inducers to tumor sites, minimizing systemic toxicity while enhancing efficacy (62). These systems can be further developed to co-deliver ferroptosis inducers alongside chemotherapeutic agents or immune checkpoint inhibitors, providing a synergistic, multidimensional approach to lung cancer treatment. Additionally, overcoming resistance mechanisms, such as tumor adaptations that evade ferroptosis by altering antioxidant pathways or lipid metabolism, will be critical. Finally, the application of artificial intelligence in ferroptosis research promises to accelerate drug discovery, optimize treatment protocols, and uncover novel therapeutic targets, paving the way for innovative and personalized treatment strategies that improve outcomes for lung cancer patients (63).

### 4.6 Limitations

This study represents a pioneering effort to analyze global research trends and potential frontiers in ferroptosis research for lung cancer through a bibliometric approach. However, certain limitations should be noted. The analysis was based exclusively on English-language articles and reviews retrieved from the WoSCC. This reliance on a single database may have excluded relevant studies indexed in other databases such as Scopus or PubMed, or publications in other languages, thereby potentially limiting the comprehensiveness of the findings. Future studies should consider integrating data from multiple databases to ensure a more comprehensive analysis. Additionally, the selection of keywords was guided by quantitative thresholds, it is important to acknowledge that the process may still involve some subjectivity, particularly in the exclusion of irrelevant terms. Another issue is that articles are influenced by their publication duration; recently published high-quality studies may have been underrepresented due to lower citation frequencies. Furthermore, bibliometric metrics, such as citation counts, reflect visibility rather than the intrinsic quality or clinical relevance of research, highlighting the need for more nuanced evaluation methods. Despite these constraints, the trends and insights elucidated in this study provide a robust foundation for understanding the current research landscape and guiding future investigations in this rapidly evolving field.

## 5 Conclusion

This study provides a comprehensive bibliometric and visualized analysis of ferroptosis research in NSCLC, highlighting global trends, collaboration patterns, and emerging research directions. Over the past decade, ferroptosis has become an increasingly prominent area of study, with significant contributions from China and the United States. Research hotspots include ferroptosis mechanisms, oxidative stress, immunotherapy, biomarkers, and nanomedicine, with recent trends emphasizing the integration of ferroptosis inducers with immune checkpoint inhibitors and advanced drug delivery systems. Our findings underscore the importance of ferroptosis in overcoming therapeutic resistance and advancing precision medicine for NSCLC. Future research should focus on unraveling the molecular mechanisms of ferroptosis, exploring its interplay with the tumor microenvironment, and addressing resistance mechanisms. International collaborations and the application of cutting-edge technologies such as nanomedicine and artificial intelligence are critical for maximizing the therapeutic potential of ferroptosis in lung cancer. Despite its limitations, this study offers valuable insights into the evolving landscape of ferroptosis research and its translational potential for improving patient outcomes in NSCLC.

## Data Availability

The original contributions presented in the study are included in the article/supplementary material. Further inquiries can be directed to the corresponding authors.
